# Analysis of Drought and Flood Variations on a 200-Year Scale Based on Historical Environmental Information in Western China

**DOI:** 10.3390/ijerph19052771

**Published:** 2022-02-27

**Authors:** Yinge Liu, Yanjun Wen, Yaqian Zhao, Haonan Hu

**Affiliations:** 1 Key Laboratory of Disaster Monitoring and Mechanism Simulating in Shaanxi Province, College of Geography and Environment, Baoji University of Arts and Sciences, Baoji 721013, China; wenyanjun2003@163.com (Y.W.); kuangbiao_2009@163.com (H.H.); 2 Institute of Water Resources and Hydro-electric Engineering, Xi’an University of Technology, Xi’an 710048, China; yaqian.zhao@ucd.ie; 3 UCD Dooge Centre for Water Resources Research, School of Civil Engineering, University College Dublin, Belfield, D04 V1W8 Dublin, Ireland

**Keywords:** historical environmental information, droughts and floods, reconstruction, influencing factors

## Abstract

Historical environmental evidence has been characterized by time accuracy, high spatial resolution and rich information, which may be widely used in the reconstruction of historical data series. Taking the upper reaches of the Weihe River as an example in Western China, the grades and index sequences of the drought and flood disasters from 1800 to 2016 were reconstructed based on various historical environmental information and standardized precipitation indicator (SPI). Moreover, the characteristics of droughts and floods were analyzed using statistical diagnostic methods, and the mechanisms affecting centennial-scale droughts and floods were discussed. The validity of reconstruction sequence of droughts/floods was verified, which showed that the reconstruction sequence may reasonably indicate the status of drought and flood. The reconstruction indicated the following periods of drought/flood: a period of extreme and big droughts in 1835s–1893s, 1924s–1943s and 1984s–2008s, a period of extreme and big floods in 1903s–1923s, and a period of extreme and big droughts/floods in 1944s–1983s. Moreover, the droughts were more serious in the western part of this region and the floods were relatively severe in the east of this region, while the droughts and floods have long-term period of about 100 years and mutation. The influence mechanism of external environmental forcing factors driving floods/droughts were revealed. The results showed that the cycle of El Niño Southern Oscillation (ENSO) and sunspot activities were closely related to the variations of drought/flood, meanwhile, ENSO has a significant lag time scale cumulative influence on droughts and floods, especially the 15-year sliding effect was the most obvious. In the peak year of sunspots, the probability of heavy drought/extreme floods was large, and the 102-year sunspot cycle has a more significant effect on drought and flood disasters. The mutation of droughts and floods occurred in the context of the drastic changes in the ground environment, and transformation of precipitation and land use structure. These results will enhance the understandings of historical environmental climate characteristics and mechanisms over the hundred years, and be useful for the future regional water resources and assessment, and ecological environment management.

## 1. Introduction

With the acceleration of global change, climate change has been the focus of scholars. The continuous rise in global temperature has accelerated the dramatic changes in the temporal and spatial distribution patterns of atmospheric circulation and hydrological cycles [1,2,3], thus causing the structural changes of the hydrological cycle system and exacerbating the occurrence of regional extreme hydrological events (such as droughts, heavy rains and floods) [4]. The International Past Global Change Research Program (PAGES) proposes to strengthen the reconstruction of global and regional scale climate integration research [5]. Historical climate over the past century has become one of the important contents of climate variability and predictability plans. At present, proxy evidence for reconstructing the historical climate can be summarized as historical documentation and information on the natural environment. According to the recorded content, it can be roughly divided into weather records, meteorological disaster records, phenology records, regional climate characteristics and their impact records, etc. [6,7]. The natural proxy evidence mainly includes tree rings, ice cores, stalagmites, sporopollen, corals, lake and bay sediments, etc. [8,9,10]. The dating accuracy, temporal resolution and spatial representation of various proxy evidences vary, and these proxy evidences should be inverted into climate information that can be linked and compared with data from instrumentation, reanalysis and simulation. At the same time, significant progress has been made in this research field using integrated multi-generation evidence to reconstruct the surrogate data sequences of the northern hemisphere and global high-resolution climate changes for more than a thousand years. For example, through the reconstruction of the historical climate, the multi-scale characteristics of long-term climate change are revealed, including natural variability and genetic mechanisms and the impact of human activities on the climate [11]. The laws of the climate system on the scales of years, decades, centuries, and even millennia have been identified [12]. The temporal and spatial characteristics of the long-term climate change periodicity, abrupt change and extreme events, as well as their variability mode and teleconnection detection are analyzed [13]. Due to the different types of regional proxy data, there are obvious differences in the indicated temperature and precipitation indicators, reconstruction methods and calibration methods. This has enhanced the understanding of climate change and hydrological cycle, and flood mechanisms over the millennium [14]. Due to the short-time nature of the modern observational data, the details of long-term climate changes and its causes were far from well understood. Therefore, the approaches to acquire the quantitative and semi-quantitative paleo-environmental climate data with high precision and high resolution have become an important technical means for the in-depth study on the changes of the historical climate and hydrological cycle. The use of environmental information (i.e., tree rings) to extract climate and hydrological elements has become the one of the most popular technical means to reconstruct regional temperature, precipitation and runoff [15]. While the historical documentation has become an important tool for reconstructing the paleoclimate and has been used in the analysis of flood disasters in different regions in 2000 [16]. Although some achievements have been made in the quantitative estimation of climate changes, it is still in the stage of development and exploration. Due to lack of early data, there was limited research and analysis on historical floods and droughts caused by precipitation anomaly. It is of great significance for the integration of multi-source data to analyze the law of climate change within a hundred years and to explore the forcing factors that affect the climate changes. When the historical meteorological data with high quality and long series can be obtained, the regional drought and flood events with high resolution can be reconstructed. Therefore, in this paper, taking the upper reaches of the Weihe River in Western China as an example, the series of droughts and floods since 1800 are reconstructed, the variation trends of droughts and floods in the study area are analyzed, with the relationship between the mechanisms of drought and flood discussed. The results obtained from this study provide a basis for understanding the regional hydrological cycle, as well as water resource assessment and ecological environment management.

## 2. Materials and Methods

### 2.1. Materials

**Figure 1:**
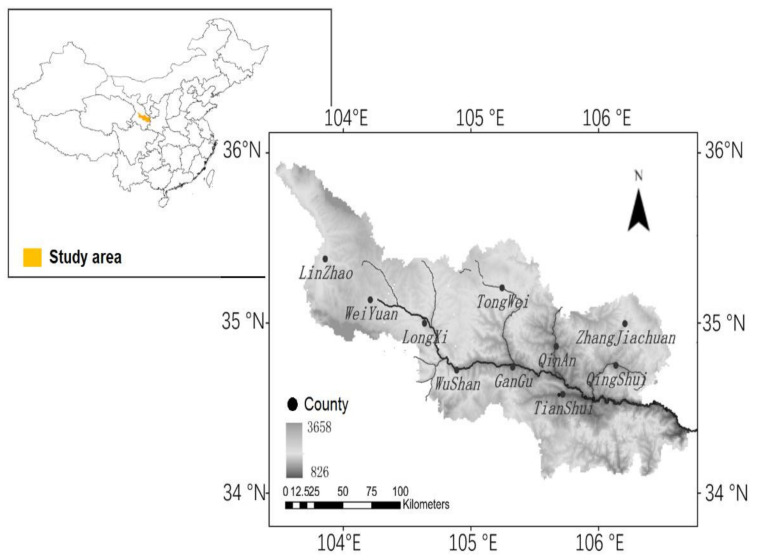
Distribution of the sample in upper reaches of the Weihe River Basin in China.

The upper reaches of the Weihe River Basin are located at latitude 34° N–36° N and longitude 103°24″ E–107°10″ E in Gansu Province, western China, covering 16 counties with areas of 25,790 km^2^, which belong to the continental semi-arid climate with little annual average precipitation of 353–547 mm and large evaporation (Figure 1). Affected by the Pacific subtropical high, the temperature is high and precipitation is concentrated with rainstorms in summer and autumn. Due to climate warming and human activities, regional hydrological runoff has been greatly reduced, and the frequency and intensity of extreme hydrological events have increased, which causes a large impact on the water resources and the economic development.

The data used in this study (1800–1950) were derived from the local chronicles and the history documentations, including: an atlas of the drought/flood category for nearly 500 years in northwestern China [17], a compendium of Chinese meteorological records of the last 3000 years and an atlas of the drought/flood category for the last 500 years in China [18,19], complete book of meteorological disasters in China (Gansu volume) [20]. The data (1951–2016) were derived from the climate bulletin made by each city and the climate impact assessment of the year and meteorological stations precipitation records.

### 2.2. Method

At present, the methods of sequence reconstruction for the paleoclimate, hydrological elements and drought and flood disasters can be summarized as follows: paleoenvironmental information, such as tree ring cores, cave stalagmites and sediments, photo-luminescence dating and historical documentation. The first few methods were limited by environmental conditions and it was difficult to obtain samples. Historical literature records avoid these shortcomings, and have the characteristics of accurate time, high spatial resolution and rich information. They were widely used in the reconstruction of historical climate data. China’s rich historical environmental documentation was an important source of paleoclimate data in the past climate sequence reconstructions when the direct high-resolution indicators were not available. Thus, we reconstructed the drought and flood sequence based on documented statistics, and took the following steps.

First, we applied the statistical evaluation of historical descriptions (i.e., typical terms) about drought/flood situations from local chronicles and bulletin with other historical documents to measure the drought/flood grade series of each site during 1800–1950 (i.e., the period without instrumental measurement records). While the grades in 1951–2016 (i.e., the period of instrumental measurement records) were converted according to the measured rainfall of 20 weather stations nearby.

According to the national standards for meteorological drought rating of China (GB/T 20481-2006) in the literature [21], the water conservancy industry standards of the republic of China—flood disaster assessment standards (SL 579-2012) in the literature [22,23] as well the precipitation indicator SPI standardized in the literature [24], combined with the actual situation of the region [25], a 7 level grading system was employed to describe the regional precipitation status, ranging from extreme drought (I) to extreme flood (VII), and the grade descriptor was presented in Table 1. The drought and flood grade standards were as follows:

(1) Grade  I           DHSP≤−0.84+0.17σ           Extreme drought

(2) Grade  II     −0.84≤DHSP≤−0.72+0.24σ          Big drought

(3) Grade  III        −0.72≤DHSP≤−0.56+0.32σ           Drought

(4) Grade  IV        −0.56+0.32σ≤DHSP≤+0.56+0.32σ   Normal

(5) Grade  V            +0.72≥DHSP≥+0.56+0.32σ           Flood

(6) Grade  VI         +0.84≥DHSP≥+0.72+0.24σ          Big flood

(7) Grade VII            DHSP≥+0.84+0.17σ           Extreme flood

(8) $$DHSP=(P-\bar{P})/\bar{P}$$

where DHSP represents the proportion of drought and flood grade anomalies, P represents reconstructed drought and flood grade of the *i*-th year, $\bar{P}$ represents annual average reconstructed drought and flood grade in 1961–1990 and σ represents standard deviation.

It was worth noting that in this study, due to the non-uniformity of the information obtained from different data and the spatial and temporal distribution, the determination of drought and flood grades in the entire study area needed to rely on the comprehensive assessment of the individual sites in the region. When defining the disaster level, the degree of droughts and floods at a single site and the types of disasters occurring at multiple sites in the same area need to be considered, at the same time, it is necessary to combine the actual situation of the Weihe River area. After the determination of the single-point drought and flood level, the regional drought and flood index was calculated, then the annual drought and flood grading value was evaluated. Accordingly, the formula for calculating the flood and drought index was established as follows.

(9) $$dfi=\sum_{i=1}^{m}\sum_{j=1}^{n}N_{j}G_{i}/\sum_{j=1}^{m}S_{j}$$

where, dfi was drought and flood index, $G_{i}$ was i-th drought and flood level, i was the number of drought and flood level, j was the number of drought and flood areas, $N_{j}$ was the j-th drought and flood level and $S_{j}$ represents all of the drought and flood areas.

Finally, we reconstructed the monthly drought and flood sequence for each station, and then established a sequence of four seasons to reconstruct the annual drought and flood sequence.

Wavelet analysis is a multi-scale analysis tool with time-frequency multi-resolution function. It is widely used in signal processing, image processing, pattern recognition, speech analysis, quantum physics and many nonlinear science fields. The series of drought and flood disasters are nonlinear, and have multi-scale characteristics in the time and space domains, and there are complex nonlinear interactions between scales. Therefore, the wavelet analysis was applied to analyze the multi-scale cycle of droughts and floods, and the sliding *t*-test was employed to identify the abrupt points of drought and flood. The correlation analysis method was used to explore the mechanism of drought and flood. The Origin, Matlab, DPS and ArcGIS software were used to process and analyze the data. The least square method was used to calculate the drought and flood trend rate, and was calculated as follows:

(10) $$TR=(\sum_{i=1}^{m}g_{i}t_{i}-\frac{1}{n}\sum_{i=1}^{m}g_{i}\times \sum_{i=1}^{m}t_{i})/[\sum_{i=1}^{m}t_{i}^{2}-\frac{1}{n}{\left(\sum_{i=1}^{m}t_{i}\right)}^{2}]$$

where, $g_{i}$ indicated the sequence of droughts and floods in the ith year, $t_{i}$ indicated time (year), m indicated the number of samples. TR indicated the trend rate, and TR>0 indicated an increase in droughts/floods, and vice versa.

## 3. Results

### 3.1. Reconstruction of Drought and Flood Sequences

**Figure 2:**
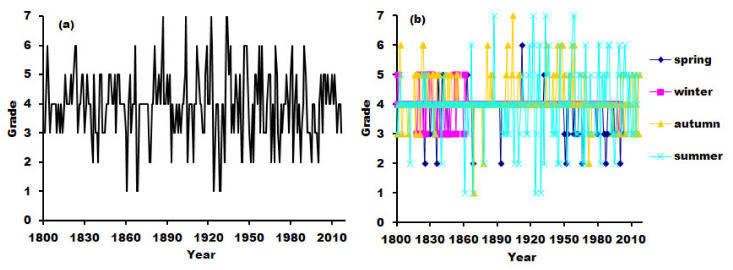
Sequences of droughts and floods in the upper reaches of the Weihe River Basin during 1800 to 2017. (**a**): Year; (**b**): four seasons.

**Figure 3:**
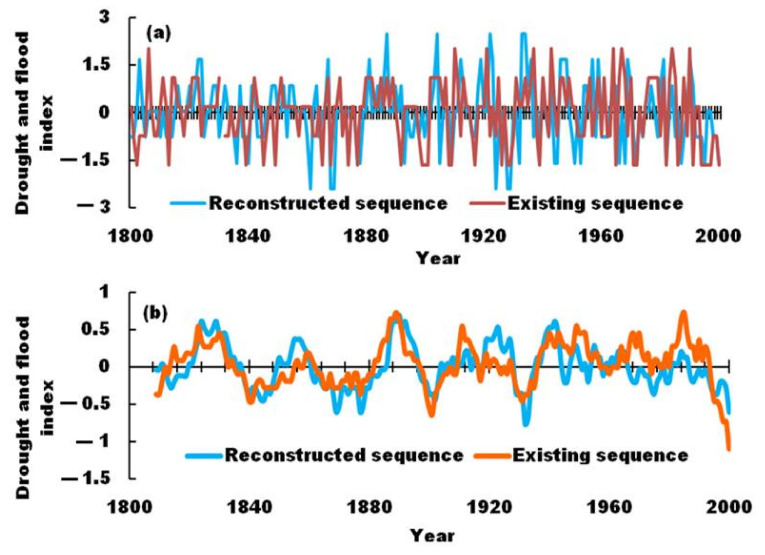
Comparison of reconstructed drought and flood sequences in the upper reaches of the Weihe River Basin with existing drought and flood sequences. (**a**): Original sequence; (**b**): 10-year sliding sequence.

The result was presented in Figure 2. The series of droughts/floods in pre-instrumental periods (i.e., 1800–1950) were continuous and consistent with instrumental periods (i.e., 1951–2016). To verify the validity of the reconstruction sequence of droughts/floods, we compared the consistency of our reconstructed sequences with the results of the literature [24] in Figure 3. The correlation coefficient between the reconstructed sequence and the existing sequence reaches 0.632 (*n* = 200, *p* < 0.001). The statistical results were basically the same, and the strong correlations demonstrates that the reconstruction sequence may reasonably indicate the status of droughts and floods. The drought and flood index since 1950 was weaker than the existing index. This may be caused by differences in the reconstruction method standards.

### 3.2. Variation of Droughts and Floods

**Figure 4:**
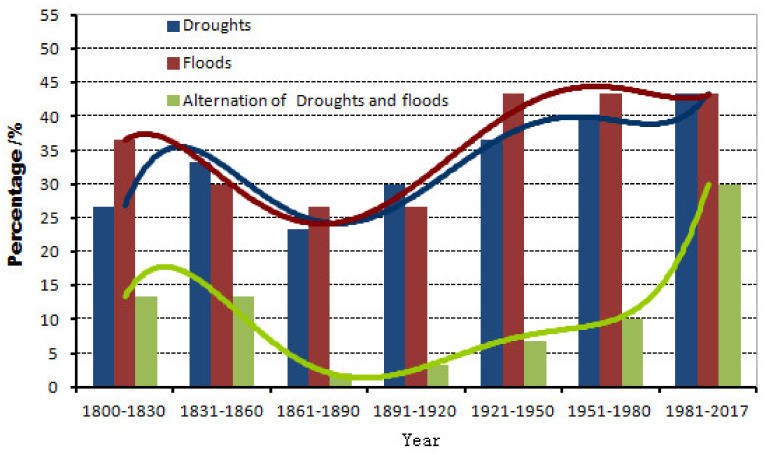
Frequency of droughts and floods in different ages in the upper reaches of the Weihe River Basin in China.

Statistics showed that over the past 200 years, there have been 70 droughts and 75 floods, of which 23 have occurred alternately. Moreover, the frequency of the droughts that occurred in spring and winter was 27 and 16, respectively, and that of the floods caused by heavy snow was 5 and 8, respectively. While the number of occurrences of droughts and floods in summer were 46 and 36, and that in autumn was 19 and 47, respectively. The frequency of droughts and floods was calculated according to different time periods in Figure 4. At the same time, Extreme and big drought/flood years were identified in Table 2. As can be seen, the frequency of droughts and floods in different periods was presented. In 1800s–1890s, drought and flood decreased, and then increased. While the frequency of extreme and big floods was the greatest in 1901s–1950s, and the frequency of extreme drought was the greatest in 1951–2000. It was a period of extreme and big drought in 1835s–1893s, 1924s–1943s and 1984s–2008s, a period of extreme and big floods in 1903s–1923s, and a period of extreme and big drought/flood in 1944s–1983s, while it was a period of continuous extreme drought in 1863–1883. From 1800 to 2016, there were four consecutive extreme droughts, four consecutive extreme floods and two alternate droughts and floods.

**Figure 5:**
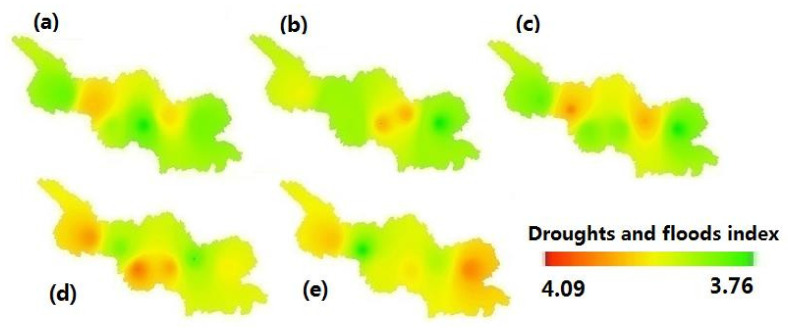
Spatio-temporal distribution of drought and flood trend rates in the upper reaches of the Weihe River Basin in China (**a**):1800s–1850s; (**b**):1851s–1990s; (**c**):1991s–1950s; (**d**):1951s–2000s; (**e**): 2001s–2016s.

Using ArcGIS kriging interpolation, the spatial distribution of drought and flood trend rates was presented in Figure 5. It showed that drought increased in the first half of the 19th and 20th century, and then the floods increased in most areas of this region. The drought trend rate was higher in the west than in the east. The maximum of floods was in Longxi and Zhangxian, and the maximum of drought was in Dingxi and Huining. Flooding was severe in the southeast and severe drought in the northwest in the upper reaches of the Weihe River Basin, China.

**Figure 6:**
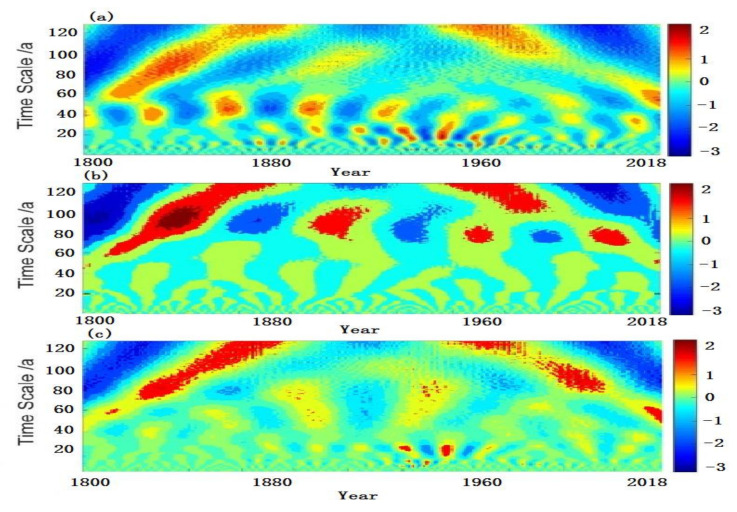

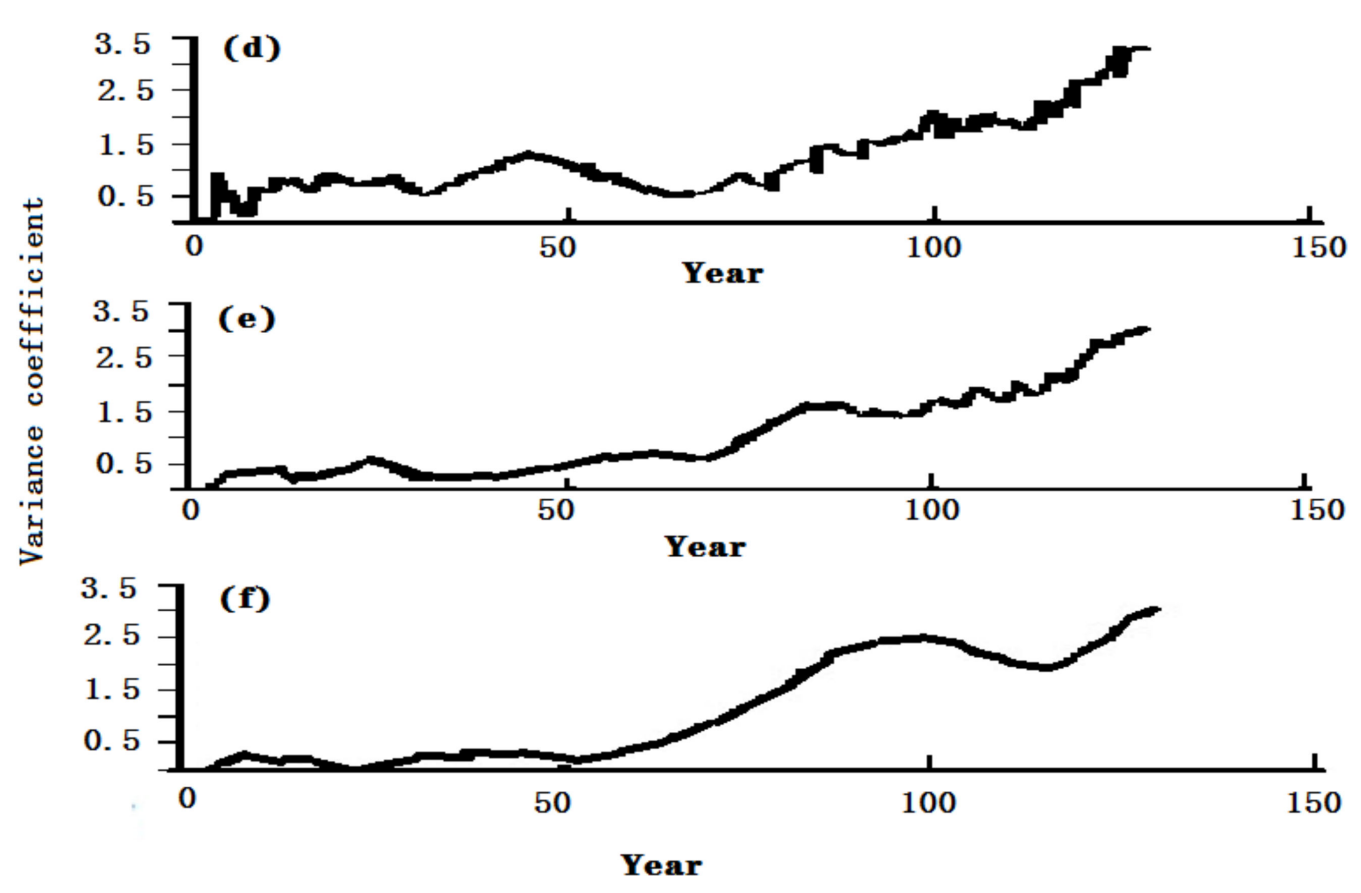
Real part and variance changes of wavelet coefficients of droughts and floods. (**a**): year; (**b**): autumn; (**c**): summer; (**d**): year variance; (**e**): autumn variance; (**f**): summer variance.

**Figure 7:**
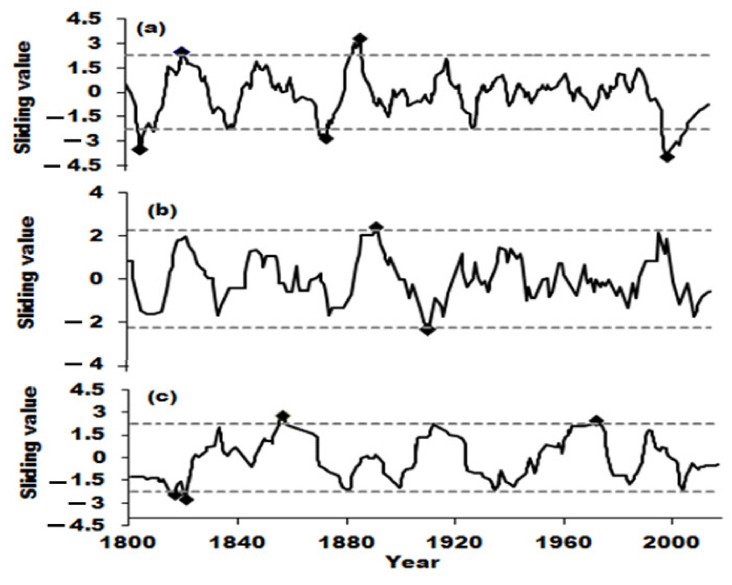
Sliding t-test for the mutations of droughts and floods during 1800–2017. (**a**): Year; (**b**): Summer; (**c**): Autumn.

The period of droughts and floods was analyzed by using wavelet, and the real part and variance of the annual drought and flood wavelets was shown in Figure 6. As can be seen, the annual droughts and floods fluctuated with periods of 11 years, 18 years, 45 years and 105 years, and showed the chronological cycle of 8 years, 42 years and 100 years in autumn and 25 years, 60 years and 110 years in summer. The 105-year cycle was even more significant, which passed the significance test at the 99% confidence level. A sliding t-test was applied to detect the mutations of droughts and floods over the past 200 years. As shown in Figure 7, it can be seen that there are more mutations in the year and autumn, and fewer mutations in summer. There were fivev mutations in the annual change, which occurred in 1805, 1821, 1875, 1887 and 2002, respectively. There were four mutations of droughts and floods in autumn, namely, in 1814, 1820, 1856 and 971, separately. In addition, two mutations occurred in summer, that was in 1889 and 1908, and droughts and floods had no mutations in winter.

## 4. Discussions

### 4.1. Droughts and Floods with Precipitation and Human Activities

The above results indicated that the droughts and floods in summer and autumn occurred frequently, especially in the autumn, which was related to the weather system changes in this basin. A large amount of precipitation in summer and autumn and the precipitation variability caused by the monsoon climate, which were prone to drought and flood. Heavy rain in summer and even rain in the autumn occurs frequently, which was likely to cause flooding. The periodic correlation between drought and flood changes and precipitation time series was analyzed, which showed that the two periods are consistent. These were consistent with the findings of Hu et al. [26]. Due to less precipitation in winter, droughts occur frequently. In the cold and dry periods of the Little Ice Age (i.e.,1812–1876) [27], the incidence of drought was relatively large, and in the 1944s–1983s extreme drought and flood events occurred most frequently owing to climate change. The floods in winter in the 19th century were mainly caused by heavy snowfall.

The increase in droughts and floods in the 20th century was related to the environmental impact of human activities. Global changes showed an obvious impact on the global hydrological historical environmental evidence cycle process, the interannual and intra-annual changes in precipitation were further accelerated and the variability of precipitation increased significantly [28]. Thus, the probability of occurrence of hydro-meteorological extreme drought and heavy rain was also elevated. According to the present study, it was found that the frequency of droughts increased after 1900, which was related to the changes in the environment and climate [29,30]. Frequency mutations were also found in the fall and within a year, which indicated that climate change was very sensitive during this period. However, there were fewer mutations in summer, winter and spring, which revealed that climate change was relatively stable. This was because this area was a sensitive area for dry and wet climate, and the mutation occurred in the context of the transformation of the global climate.

**Figure 8:**
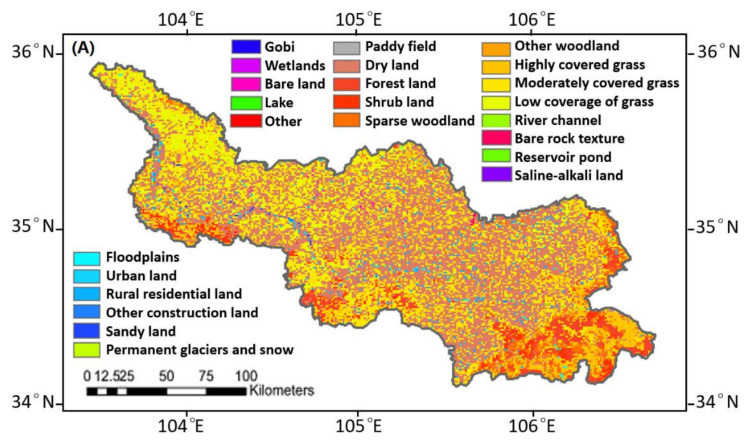

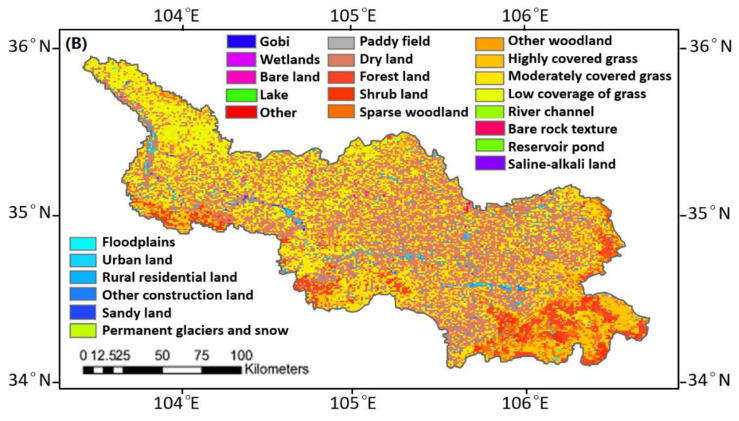
Land use in the upper reaches of the Weihe River Basin in 1980 (**A**) and 2015 (**B**).

Moreover, the possibility of droughts and floods was increased due to the change of catchment and flow of the river basin and the convergence process of the river channel, caused by land use, water conservancy projects and river basin remediation, as shown in Figure 8. Land use structure in the study area has undergone tremendous changes from 1980 to 2005, with cultivated land and wetlands and lakes reduced by 2.6% and 1.9%, and the construction and transportation land increased by 1.7%. Due to the strengthening of human activities, the river dams in the Weihe River Basin have been severely damaged, which led to serious soil erosion, increased evaporation and increased surface runoff, thus causing the frequency of droughts and floods.

### 4.2. Droughts and Floods with ENSO

**Figure 9:**
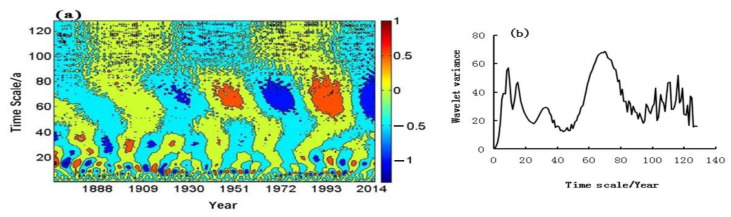
Wavelet analysis of ENSO. (**a**): wavelet real part of ENSO; (**b**): wavelet variance of ENSO.

**Figure 10:**
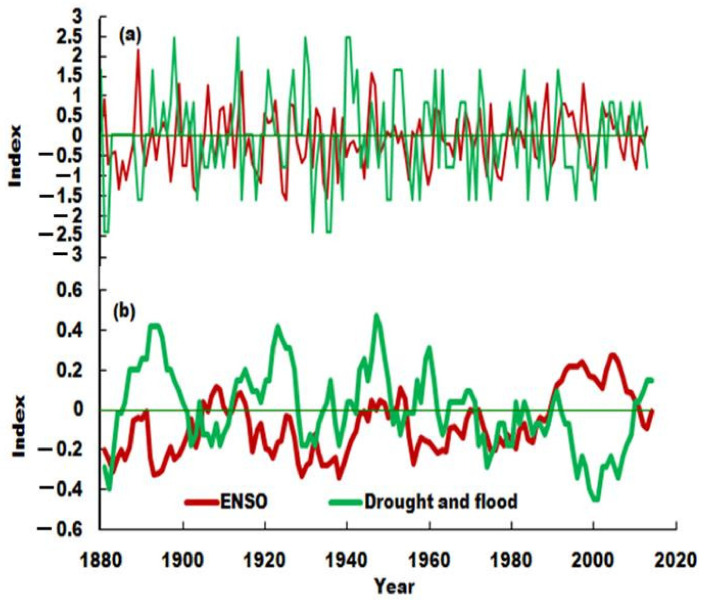
Comparison of ENSO and drought and flood index. (**a**): original sequence; (**b**): 15 years sliding sequence.

The high frequency periodic variation of ENSO has been considered an important signal for climate impact [31]. As can be seen from Figure 9, ENSO has a long period of 7–10 years, 15–18, 70 and 102–105 years. The main ENSO cycles were consistent with periods of droughts and floods of the upper mainstream of the Weihe River. Studies have pointed out that there was a certain teleconnection between El Niño and precipitation in northwestern China [32,33], which was more obvious in the summer and autumn, when ENSO occurred. Our results verified the multi-decadal teleconnection between drought/floods in the Weihe River Basin and ENSO by a negative correlation. To further verify this multi-decadal teleconnection relationship, the 15-year sliding correlation coefficient for ENSO and droughts/floods were calculated, and the coefficient was −0.457 (*n* = 118, *p* < 0.001), and the relationship between ENSO and droughts and floods was significant in Figure 10. It can be seen that the periods of strong/weak ENSO were coincident with the troughs of droughts and floods. It was revealed that ENSO has a significant lag time scale cumulative influence on droughts and floods. During the strong ENSO period, the drought was severe, and in the weak ENSO period, the frequency of flooding was high, and vice versa. It was believed that when the ENSO phenomenon occurred, the surface temperature of the sea surface in the eastern equatorial Pacific rose, and the temperature gradient increased, the Hadley circulation increased, resulting in an increase in the intensity of the western Pacific subtropical high, thus affecting precipitation in northwestern China. Thus, the occurrence of droughts and floods in the Weihe River may be affected by the periodic ENSO.

### 4.3. Droughts and Floods with Sunspots

**Figure 11:**
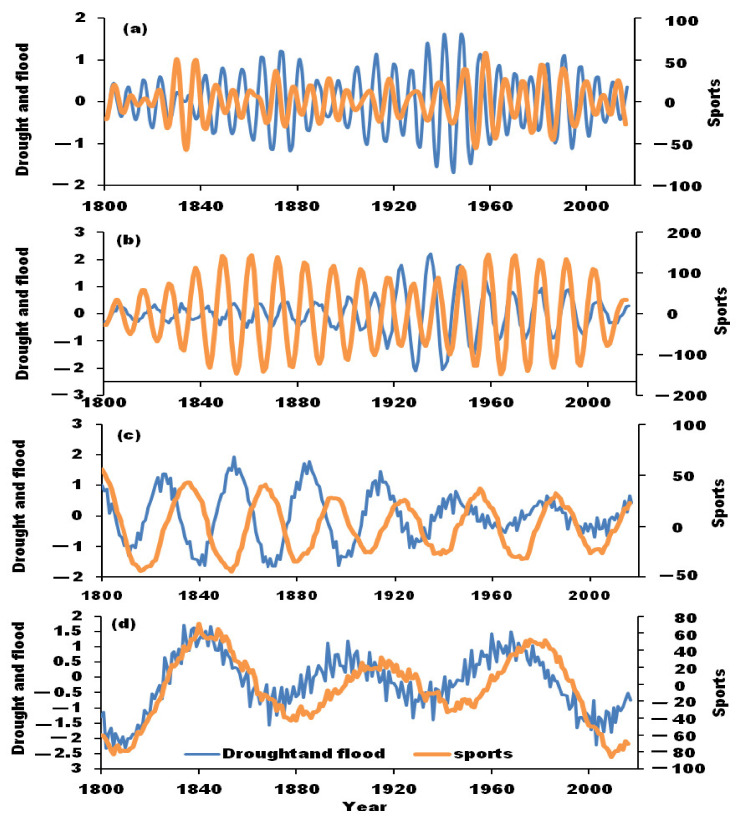
Correlation of droughts/floods and sunspot activities component from 1800 to 2017. (**a**): 11-year cycle component; (**b**): 18-year cycle component; (**c**): 45-year periodic component; (**d**): 102-year periodic component.

The impact of solar activity on the climate has attracted people’s attention as early as the 17th century [34]. Moreover, some significant inter-decadal solar activity signals were usually detected in climate anomalies in specific areas [35,36]. Our study found that droughts and floods have long periods and short periods, and the extreme events of droughts and floods in the upper reaches of the Weihe River were also related to the cycle of sunspot activities (Figure 11). It can be clearly seen that in the 11-year periodic component change, the droughts and floods that occurred before 1880 were positively correlated with sunspot activities, then, they were inversely correlated. After 1920, the correlation between these disasters and sunspot activities was positive again. In the 18-year and 45-year cycle component change, there was an anti-correlation between these disasters and sunspot activities before 1920, which then became positive. In addition, in the 102-year cycle change, the results showed that droughts and floods exhibited a significantly positive correlation with sunspot activities, indicating the latter had an important impact on the cycle of droughts and floods, especially after 1920, which indicated that the climate system in this region has a sensitive mechanism for the response to the solar activity cycle. According to statistics, the sunspot peak year corresponded to the extraordinarily large drought years, namely 1861, 1868, 1869, 1824, 1930 and 1929, and the sunspot valley year corresponded to the heavy flood year, namely 1887, 1904, 1922 and 1934. Generally, the relatively low-intensity period of sunspots corresponded to the flood disasters, and the relatively high-intensity period corresponded to the occurrence of drought disasters.

From the results, the climate sequence reconstructed by historical environmental information can better analyze the temporal and spatial characteristics of droughts and floods in the Weihe River Basin, and these research results are basically consistent with the literature [4,25]. At present, the proxy evidence for reconstructing the historical climate includes historical document records and natural proxy evidence. The use of historical document records, loess, ice cores, ocean and lake sediments, tree rings, corals and stalagmites to obtain climate and hydrological information is an important tool of regional hydrology and paleontology [37,38]. Based on the weather records, meteorological disaster records, phenological records, regional climate characteristics and their impact records, this study quantifies the drought and flood grades, analyzes the influencing factors of the droughts and floods in the Weihe River Basin, and finds that droughts and floods are closely related to ENSO and sunspots. These findings are consistent with the literature [32]. However, due to the differences in the methods of quantitative reconstruction of drought–flood sequences and the degree of quantification, the results may be different. Therefore, the combination of multiple methods and the extraction and synthesis of multi-source environmental information need be further studied.

## 5. Conclusions

The reconstructed sequence may reasonably indicate the status of droughts and floods based on various historical environmental information and descriptions. The droughts and floods in the upper reaches of the Weihe River showed the characteristics of interannual variation. In the 18th century, droughts were more severe in this river basin. However, after the 19th century, floods in this basin gradually increased, and the frequency of droughts and floods showed a rising trend, especially the frequency of extreme events of droughts and floods in the 1944s–1983s. There were spatio-temporal differences between the droughts and floods that occurred in the upper reaches of the Weihe River. The droughts were more serious in the western part of this region and the floods were relatively severe in the east. At the same time, the droughts and floods in this basin also exhibited the characteristics of long-term and short-term variations and mutations. The strongest period of oscillation was 105 years.

The frequency of the droughts that occurred after 1900 increased, which was related to the changes in the environment and climate. Land use structure has undergone tremendous changes, thus causing the frequency of droughts and floods. Moreover, ENSO showed a lagging cumulative influence on droughts and floods. The droughts corresponded to the strong ENSO period, and the floods corresponded to the weak period of ENSO. The periods of sunspot activities were closely related to the variations of drought and flood cycle components, especially the sunspots 11-year period was significantly positively correlated with the heavy droughts/floods. In the peak year of sunspots, the probability of heavy droughts was high, and in the low year of sunspots, the probability of extreme floods was large. Externally forced environmental factors have an important impact on flood/drought disasters.

Historical documents have the advantages of accurate time, high spatial and temporal resolution and rich information among various evidence of climate and environmental changes in historical periods. However, the time coverage of historical documents depends on the start time of historically written records in various places, which has the defect of incomplete records. How to solve the shortcomings of omissions and omissions in ancient meteorological records, and the quantification and standardization of historical document records are the keys to improving the precision and accuracy of paleoclimate sequences.

## Figures and Tables

**Table 1:** Classification standard of droughts and floods.

Grade	Descriptions	Level of Droughts and Floods
VII	Heavy rains in many places have led to farmland flooding and river flooding	Extreme flood
VI	Continued rains and the rains caused water stagnation, sudden mountain torrents, and river dam collapse, thus destroying farmland and causing soaring food prices.	Big flood
V	The rains did not damage the residential houses. In addition, it showed less impact on crops and people’s production and life.	Flood
IV	No drought or disaster was recorded.	Normal
III	Slight lag or delay of rain which slightly impacted on crops.	Drought
II	No precipitation in successive months, which caused the river to dry up, and food prices to rise.	Big drought
I	There was no precipitation in a wide range of quarters, which caused crops to fail.	Extreme drought

**Table 2:** Extreme and big drought/flood years identified in the upper reaches of the Weihe River Basin in China.

Period	Extreme and Big Drought Years	Extreme and Big Flood Years	Drought/Flood Alternate Years	Continuous Drought Years	Continuous Flood Years	Number of Droughts/Floods
1800–1834		1803,1823,1824			1823,1824	0/3
1835–1862	1836,1840,1861					3/0
1863–1885	1868,1869,1877,1878	1867,1881	1867,1868	1868,1869,1877,1878		4/2
1886–1902	1893	1887				1/1
1903–1923	1905,1909	1904,1912,1919, 1922,1923	1904,1905		1922,1923	2/5
1924–1932	1924,1928,1929			1929,1929		3/0
1933–1943	1933,1934,1936				1933,1934	3/0
1944–1983	1944,1945,1951,1953 1966,1968,1992	1946,1947,1957,1959 1972,1981	1981,1982	1944,1945	1946,1947	7/6
1984–2008	1987,1995,2000	1990				3/1

## Data Availability

Not applicable.
